# New Pathways for Alimentary Mucositis

**DOI:** 10.1155/2008/907892

**Published:** 2008-09-23

**Authors:** Joanne M. Bowen, Dorothy M. K. Keefe

**Affiliations:** ^1^Department of Medical Oncology, Royal Adelaide Hospital, Adelaide, SA 5000, Australia; ^2^Division of Medicine, The University of Adelaide, Adelaide, SA 5000, Australia; ^3^Mucositis Research Laboratory, Hanson Institute, Frome Rd, Adelaide, South Australia 5000, Australia

## Abstract

Alimentary mucositis is a major dose-limiting toxicity associated with anticancer treatment. It is responsible for reducing patient quality of life and represents a significant economic burden in oncology. The pathobiology of alimentary mucositis is extremely complex, and an increased understanding of mechanisms and pathway interactions is required to rationally design improved therapies. This review describes the latest advances in defining mechanisms of alimentary mucositis pathobiology in the context of pathway activation. It focuses particularly on the recent genome-wide analyses of regimen-related mucosal injury and the identification of specific regulatory pathways implicated in mucositis development. This review also discusses the currently known alimentary mucositis risk factors and the development of novel treatments. Suggestions for future research directions have been raised.

## 1. Introduction

Recent research has indicated that cytotoxic chemotherapy causes unwanted normal tissue damage via its effects on a multitude of cellular regulatory pathways [1, 2]. In particular, investigations have begun to elucidate the role of regulatory pathway activation and suppression in regimen-related gastrointestinal toxicity [2, 3]. Mucosal toxicity following anticancer treatment is clinically referred to as mucositis. Mucositis was once separated into oral and gastrointestinal, however it is now widely known as alimentary mucositis, referring to the damage which occurs along the entire orodigestive tract. Alimentary mucositis affects the mucosa, causing mouth and throat pain, ulceration, abdominal pain, bloating, vomiting, and diarrhoea depending on the target tissue [4–6]. The general mechanisms underpinning the development of mucositis are thought to be the same regardless of the location along the length of the tract. However, the kinetics of symptom development is different in each region, which is thought to reflect the local turnover rate of epithelial cells and specialised differentiation for local function [4, 7]. The frequency of alimentary mucositis varies depending on the cancer and treatment, ranging from 10–40% in patients undergoing standard chemotherapy for solid tumours, up to 60–100% in those undergoing high dose conditioning chemotherapy for stem cell transplantation [5, 8].

## 2. Burden of Alimentary Mucositis

Alimentary mucositis represents a significant clinical and economic burden in oncology. The presence of any mucositis during a cycle of chemotherapy significantly increases the risk of dose reduction, the frequency of infections and bleeding, and increases the length and cost of hospitalisation. Reduction in treatment doses leads to reduced survival [9], and mucositis is also a risk factor for mortality due to its association with infection [10]. What is more, severe mucositis has been shown to be a significant risk factor for inferior overall survival, relapse mortality, and nonrelapse mortality in specific settings [11]. Resource utilisation for patients during episodes of mucositis is also significantly increased, with the need for nutritional adjuncts including fluid replacement, liquid diets, and total parenteral nutrition. Due to the association with infection, antibiotic therapy is also more common in patients with mucositis. Combined, this translates to an incremental cost increase of US$3500 per cycle of standard dose chemotherapy with oral mucositis [12, 13] and US$6000 with both oral and GI mucositis [14]. While the full burden of mucositis has yet to be defined, a prospective study is ongoing to determine its true quality of life and economic impact.

## 3. Current Overview Mucositis Pathobiology

Accumulating evidence has strengthened the proposed biological model of mucositis [15, 16]. It is known as the multiple mechanism model, as it represents a divergence from the initial linear view that mucositis was purely a result of cytotoxic agent-induced damage to basal epithelial cells [17, 18]. There is indeed interference with epithelial stem cell turnover and increased apoptosis following anticancer treatment, however this is but one component of the pathobiology of alimentary mucositis. Rather, the events that lead to mucosal injury are multifactorial, complex, and pan tissue and are driven initially by the submucosa via endothelial signalling [16, 19]. Epithelial breakdown represents the clinical stage only and is associated with loss of barrier integrity, host infection, and considerable pain [4, 7, 20–22]. Mucositis has been described as having 5 phases, which are overlapping and interactive [16, 22]. The 5 phases are initiation, primary damage response/upregulation and message generation, signal amplification, ulceration, and healing. Briefly, cytotoxic agents initiate damage through the generation of reactive oxygen species which causes both direct damage to tissue components of the mucosa and also activates secondary signalling. The primary event of the message generation phase centres around activation of the transcription factor NFkB, which leads to the upregulation of many genes involved in perpetuating mucosal injury, including proinflammatory cytokines, adhesion molecules, and cyclooxygenase-2. During the third phase, a feedback loop occurs whereby the proinflammatory cytokine, TNF, acts on a number of pathways to reinforce NFkB activation and the ceramide pathway. The ulcerative phase comprises loss of mucosal integrity and bacterial colonisation, with subsequent further proinflammatory cytokine production. Alimentary mucositis is usually self-resolving once treatment ceases, and healing occurs with renewal of epithelial proliferation and differentiation and reestablishment of the normal local microbial flora [16, 23, 24]. It is evident that this current model incorporates sequential interaction between all cell and tissue types of the mucosa and submucosa, as well as tissue factors, cytokines, and elements of the luminal environment [25, 26]. Among others, the pathways implicated in this model include ceramide signalling, extracellular matrix turnover, oxidative stress signalling, apoptosis, cytokine signalling, and cell cycling.

## 4. Special Considerations for the GIT

Mucositis pathobiology has been extrapolated mainly from research in the oral setting. However there is rapidly accumulating evidence that supports the theory that mechanisms are similar along the entire length of the alimentary canal. In models of mucositis, inflammatory mediators, TNF and NFkB, have been shown to increase along the length of the gastrointestinal tract corresponding to histological damage [27, 28]. Furthermore, proinflammatory cytokines, interleukin 1 and 6, are increased following methotrexate treatment and associated with a loss of gut barrier function [29]. Disruption of these signalling pathways ameliorates intestinal mucositis. Treatment with NFkB inhibitors has been shown to partially prevent mucosal injury in an animal model of anticancer treatment [30]. Another anti-inflammatory agent under development, RDP58, significantly inhibited intestinal damage induced by irinotecan through its ability to prevent treatment-related increases in TNF, Interferon-*γ*, and interleukin-12 [31]. Research is ongoing to fully elucidate the mechanisms of gastrointestinal mucositis. Loss of crypt stem cells remains an important event in intestinal injury following anticancer treatment. However, it is not the sole contributing factor leading to overt damage. The inflammatory cascade is being realised as an important pathway in the development of intestinal mucositis that can be pharmacologically manipulated.

## 5. Overview of Current Understanding of Risk Factors for Mucosal Toxicity

Mucosal susceptibility is based on global and tissue-specific factors. Global components are associated with treatment and patient characteristics, while tissue specific components are related to epithelial type, the intrinsic endocrine system, and the local microbial environment [32]. The choice of drug, the schedule, and dose-intensity of the regimen will impact on the risk of toxicity [33]. Patient related variables include gender, ethnicity, and presence of comorbidities such as diabetes mellitus [32], although the absolute association is far less clear for these variables compared to treatment. Cellular and molecular elements that influence toxicity will depend on the local tissue environment, in which mucosal responses to damaging stimuli, whether direct or indirect through intermediate mediators, are contingent on the particular location of the tissue and epithelial type, microbial environment and specific regional functions [32].

While the interactions between global and tissue specific factors impact on mucosal injury, there are also underlying genetic influences that profoundly affect toxicity. The genetic component for predisposition to mucositis is well established [34, 35]. A classic proof of principle example is the finding from clinical trials that genetic susceptibility to apoptosis can impact on the risk of mucositis. Patients with Addison's disease, a condition characterised by excess apoptosis, are 17% more likely to develop oral mucositis during anticancer treatment, and patients with Psoriasis, a condition of reduced apoptosis especially in skin, are 77% less likely to suffer oral mucositis [15, 16].

Although this is an elegant example, the most widely accepted evidence for the genetic basis of mucositis risk is the observation that patients deficient in drug-metabolising enzymes are at a higher risk of treatment toxicity [32]. Specific examples of these include deficiencies in UDP-glucuronosyltransferase 1A1 (UGLT), methylenetetrahydrofolate reductase (MTHFR), thymidylate synthase (TS), and dihydropyrimidine dehydrogenase (DYDP), during irinotecan, methotrexate, and 5-FU treatment, respectively [34, 36]. Reduced levels of these proteins can be caused by hereditable inactivating mutations in the gene promoter region and result in accumulation and drug prolonged exposure. Pharmacogenetics seems an attractive solution to explain all mucotoxicity, however the proportion of patients with these enzyme deficiencies is greatly less than the number of patients that suffer toxicity [2, 32]. It has been suggested that genetic variants of mucositis mediators, rather than metabolism enzymes, may be associated with the majority of toxicity. One such mediator is TNF. Recent studies have shown that TNF gene polymorphisms are associated with altered risk of toxicity following cancer treatment [37, 38]. Patients that are heterozygous for the TNF-308 promoter polymorphism have increased TNF production and are at a significantly greater risk of toxicities following myloablative chemotherapy treatment. Patients had a 17-fold increased risk of complications following treatment including those affecting the oral mucosa, skin, and gut, which is a stronger association than traditional mucositis risk variables of gender, age, and treatment dose [37]. Although these initial findings are encouraging, the complexity of mucositis means that there is a continuing challenge to discover more polymorphisms within candidate genes predictive of regimen-related mucosal damage. These types of studies provide accumulating evidence to suggest that a number of genetically controlled elements can determine the response of the mucosa to cancer treatment, and that these can be either generic or tissue specific (Table 1).

**Figure 1:**
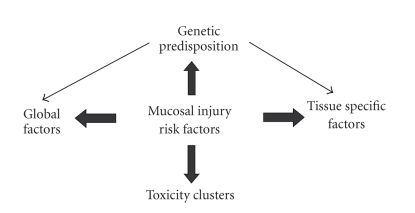
Relationship of proposed contributors implicated in development of alimentary mucositis.

Finally, the most recent area of research involved in defining risk factors for mucositis is toxicity clustering. Symptom clustering has previously been applied to a range of nonmalignant diseases, and to cancer symptoms, and has proven useful for creating diagnostic criteria. However, in the context of anticancer treatment-related toxicity, it is in its infancy [39]. The general principle is that a patient who has alimentary mucositis is likely to have a particular subset of other toxicities and vice versa. A recent paper used Bayesian analysis to identify linked toxicities [39] and Markov networks to define clusters of toxicities [40] in colorectal cancer patients following treatment. It found that gastrointestinal toxicities clustered together strongly, implying that each is a risk factor for the other and may represent a common underlying aetiology [39]. This novel work has provided the opportunity to gain a new insight into the relationship of multiple regimen-related toxicities. Overall, it seems that defining mucosal injury risk factors is as complex as the pathobiology. The major components include global, tissue specific, genetic and clustering factors, and these are combined with cellular and molecular interactions between factors which ultimately determine the mucosal response to chemotherapy treatment (Figure 1).

## 6. New Alimentary Mucositis Pathway Research

The biological events which induce alimentary mucositis begin almost immediately following administration of radiotherapy and chemotherapy [21, 41]. Inhibition of these early molecular events may have a profound impact on the intensity of mucosal damage and as such represents an attractive focus for research. Applying genomic profiling to mucositis research to investigate global molecular changes following treatment is an increasing area of interest that holds much promise. The ability to simultaneously investigate thousands of genes and their response to treatment has enhanced our ability to define the mechanisms of damage specific to each drug and the generic response of tissue to anticancer treatment. We can now hypothesis that many genes are initially affected by anticancer treatment which may be either unique to each drug or common to all drugs. However, these early genes must activate a smaller subset of downstream genes, involved in specific signalling pathways that are vital to propagating the cascade towards clinically significant mucosal damage. What is more, it is likely that a threshold of key gene activation must be reached before damage becomes inevitable.

Two recent studies have directed considerable effort towards investigating the gene expression changes and pathway activation responsible for alimentary mucositis development [2, 3]. Firstly, Sonis et al. investigated the relationship between gene expression, canonical pathways, and functional networks in peripheral blood monocytes of patients who developed mucosal injury in response to chemoradiation [2]. This study combined Bayesian theory with network analysis to define the canonical pathways most relevant to regimen-related toxicity. They prioritised 14 pathways derived from the Ingenuity Pathways Analysis Library (Table 2). The study by Bowen et al. investigated gene expression and pathway modulation in the gastrointestinal tract of rats following irinotecan treatment [3]. They analysed their gene list using web tool, Pathway Miner, which searches genes based on their associations with cellular and regulatory pathways and performs a statistical test to rank the most significant pathways [42]. They found over 20 pathways from the KEGG database involved in tissue injury following cytotoxic treatment (Table 2). In both studies, Fisher's exact test was applied to the data set to determine the most significant pathways associated with toxicity. Metabolic pathways were also highly represented in pathway lists and included nitrogen metabolism, oxidative phosphorylation, purine metabolism, prostaglandin and leukotriene metabolism, and glutathione metabolism but were not investigated further. Gene association networks are an informative method for analysing pathway relationships among genes that are coregulated and for analysing up or down-regulated pathways that contain many participating genes [42].

These papers are the first to use a bioinformatics approach to define the pathways altered by anticancer treatment during alimentary mucositis development. Despite the use of different databases in the two studies, there is considerable overlap within the prioritised pathways named. The difference between sampling methods (peripheral blood verses gastrointestinal whole tissue) makes the results even more interesting, as it shows that local tissue damage is also represented systemically. This has important implications for future research. It is also vital that future studies direct efforts towards determining which signalling pathways are true drivers of damage and which are passengers, altered without causing a functional change at the tissue level. What is more, the kinetics of pathway activation needs to be determined to elucidate which are altered early as damage initiators, which are upregulated as a consequence of damage, and which of those are crucial to healing. Each represents a separate target for intervention.

## 7. Fitting Treatments with Pathways

There are a growing number of new antimucotoxic agents currently being clinically tested. Mostly these agents target at least one or a few pathways identified as associated with mucosal injury. However, recent research indicates that only the agents that modulate multiple pathways will be truly effective at inhibiting damage. A recent Cochrane Library review of interventions for preventing oral mucositis found that of the 33 interventions studied twelve showed some evidence of a benefit. Of these, two pharmaceutical agents appear particularly promising, namely, Amifostine and Benzydamine [43]. These agents have also been included in the Updated Clinical Practice Guidelines for the Prevention and Treatment of Mucositis [44]. Amifostine is a free radical scavenger which exerts its effects by reducing direct DNA damage and reducing upregulation of inflammatory pathways [45]. It has recently been recommended for use in prevention of radiation-induced proctitis, however it has yet to be recommended for mucositis [44, 46]. Benzydamine is recommended for the prevention of mucositis in radiation patients. It has anti-inflammatory, analgesic, anaesthetic and antimicrobial effects, although its primary mode of action is thought to be through inhibition of proinflammatory cytokines [14]. Mucositis is essentially an inflammatory condition [47, 48], and the results of the microarray studies confirm this paradigm by identifying multiple signalling pathways involved in inflammation. These include NFkB signalling, complement and coagulation cascades, toll-like receptor signalling, MAPK signalling, cytokine-cytokine receptor interaction, as well as others shown in the table above. It seems that these two agents have the potential to significantly ameliorate mucosal injury through modulating a large number of regulatory pathways. Still, Palifermin (recombinant human KGF-1) remains the only agent currently approved by the FDA for the prevention and treatment of mucositis [49]. It is biologically pleiotropic. Its primary mode of action was initially thought to rely on accelerating healing by its role as an epithelial mitogen enhancing proliferation, migration, and differentiation of mucosal epithelium. However, Palifermin has also been shown to exhibit protective functions outside of its general expected role, including inhibition of epithelial cell apoptosis and DNA damage, upregulation of detoxifying enzymes, and downregulation of proinflammatory cytokines [50]. It is likely that Palifermin does this through some of the pathways discussed. In addition to these drugs, pathway knowledge could be broadly applied to the development and discovery of new agents effective in preventing mucosal injury.

## 8. Future Directions for Research

One of the major challenges in defining the most appropriate molecular targets for mucositis intervention is the highly complex and interactive nature of its pathobiology. It seems likely that the main approach to elucidating the polygenic determinants of treatment will be genomics, in particular applying the use of anonymous single nucleotide polymorphism (SNP) maps to perform a genome-wide search for SNPs associated with treatment effects [51, 52]. This is opposed to the more traditional technique of singly investigating candidate genes based on existing knowledge of a drug's mechanism of action and the known pathways of metabolism and deposition [51, 52]. This “omic” direction, coupled with powerful bioinformatics, should greatly advance our ability to unravel the complex network of mucosal response to treatment in the near future.

Since it is assumed that the body has only a limited number of possible responses to injury [46], lessons can also be learnt from other inflammatory conditions. Recurrent aphthous stomatitis (RAS) is an inflammatory disease of the oral mucosa that is characterised by an aberrant cell-mediated immune response following external stimuli. The main feature is ulcer formation precipitated by clinical or subclinical trauma [53]. The specific immune defect has yet to be identified and the pathogenesis is unknown. However, a study showed that there are elevated resting levels of proinflammatory cytokines, particularly TNF, within nonlesional mucosa of patients with RAS compared to normal controls and lower levels of the anti-inflammatory cytokine, interleukin-10, in RAS patients following trauma. Furthermore, RAS patients all develop ulcers at mucosal trauma sites, while normal patients show minimal inflammation at the same site [53]. The proposed mechanisms for this include enhanced T-cell activation together with an increased sensitivity of keratinocytes to locally derived cytokines. It was proposed to be driven by TNF, while an absence of anti-inflammatory interleukin 10 (IL-10) may mediate the enhanced and prolonged injury response [53]. More recently, a study investigating functional gene polymorphisms in people with RAS showed a marked increase in the IL-1*β* and TNF heterozygous genotypes in ulcer sufferers [54]. This indicates a genetic predisposition to ulcer formation following mucosal injury, and due to the overlapping features with regimen-related mucosal injury, the implications are that polymorphisms in inflammatory cascades will also be useful in predicting mucositis risk.

Finally, a critical point that should be addressed before any pathway-targeted treatment can be developed is the need to define differences in modulation of pathways in normal tissue that result in toxicity versus the desired effect on tumour tissue. We must be careful not to interrupt the intended purpose of chemotherapy, and therefore consideration needs to be given to designing drugs that are either specifically targeted to the mucosal surface or that exploit features of the normal cell for drug uptake.

## 9. Conclusions

Alimentary mucositis research has entered the “omic” era. With this has come a new depth of understanding of the molecular and cellular interactions associated with development of mucosal injury in response to cancer treatment. One of the most exciting outcomes of recent research has been the characterisation and prioritising of pathways implicated in mucositis pathobiology. In the future, this piece of information will help to rationally design improved drugs and provide early identification of patients at risk of developing severe mucositis.

## Figures and Tables

**Table 1:** Genetically controlled elements that may directly or indirectly influence alimentary mucositis risk.

Generic	Tissue specific
Drug metabolism, targets and transport	Trefoils
Transcription factors	Adhesion factors
Proinflammatory cytokines	Defensins
Mediators	Secretins
Susceptibility to apoptosis and rate	Differential response to CT/RT

**Table 2:** The top 14 cellular and regulatory pathways deemed to be most relevant to mucosal injury from anticancer therapy.

Rank	Chemoradiation (Sonis, et al.)	Irinotecan (Bowen, et al.)
1	Toll-like receptor signalling	MAPK signalling
2	NF-kB signalling	Cell cycle
3	B-cell receptor signalling	Complement and coagulation cascades
4	PI3K/AKT signalling	Gap junction
5	Cell cycle	Calcium signalling
6	P38 MAPK signalling	Apoptosis
7	Wnt/B-catenin signalling	Leukocyte transendothelium migration
8	Glutamate receptor signalling	VEGF signalling
9	Integrin signalling	Cytokine-cytokine receptor interaction
10	VEGF signalling	Neuroactive ligand-receptor interaction
11	IL-6 signalling	Wnt signalling
12	Death receptor signalling	B cell receptor signalling
13	SAPK/JNK signalling	T cell receptor signalling
14	T-cell receptor signalling	Focal adhesion
